# Applied Anatomy and Oral Surgery

**Published:** 1911-11-15

**Authors:** 


APPLIED ANATOMY AND ORAL SURGERY.—This is the title to a beautifully made book just off the press of W. B. Saunders Co., Philadelphia, which is edited by Robert H. Ivy, M.D., of Philadelphia. Part one is given over entirely to presenting most entertainingly all the anatomy of the head, face and neck, which would ordinarily come for treatment to the oral rather than the

Dental Surgeon. The second part is given over to a discussion of diseases which demand surgical interference, and it is unique for the concise statements, and the surgical procedures show Dr. Ivy to be a man of wide surgical experience, who has given much time and thought to the preparation of this book, which he will never have cause to be ashamed of. It is just such a manual as will appeal to the average student, and would be a splendid thing for every practitioner to have in his library for frequent references. The cost is small, only one dollar and fifty cents, and it will be worth many times this to any one who will give the little time required to consult. It is made more complete by one of the best indexes it has ever been our pleasure to consult.

